# Mortimer House, Clifton

**Published:** 1954

**Authors:** 


### Editorial

### MORTIMER HOUSE, CLIFTON

This hospital which comes under the Southmead Hospital Group is in anomalous position. It is used by the Group as a maternity hospital, but the building is the property of the Local Authority and is held by the Health Committee. By them it is leased to the Regional Hospital Board. The Bristol Health Committee have now decided that they want Mortimer House back, as they need it for pressing demands on their Mental Health Services. The Regional Board, on the other hand, feel it would be a great pity if about forty maternity beds were lost to the City when already the demand is such on the Southmead Group that patients are being sent out before the end of the normal lying-in period—such is the pressure on the maternity beds. We understand that as the Bristol Health Committee are likely to press for the expiry of the lease of Mortimer House, the Regional Hospital Board are being asked to seek powers from the Minister to build a General Practitioner Maternity Unit in the south of Bristol on land which has been allocated for a new hospital. It is unlikely that a new hospital will be established for a long time to come, but it would be a good idea if a start were made with such a maternity unit. We feel sure it would be a pity if maternity beds were lost in the City without any reasonable expectation of their being replaced in the immediate future.

